# An improved collaborative filtering method based on similarity

**DOI:** 10.1371/journal.pone.0204003

**Published:** 2018-09-24

**Authors:** Junmei Feng, Xiaoyi Fengs, Ning Zhang, Jinye Peng

**Affiliations:** 1 School of Electronics and Information, Northwestern Polytechnical University, Xi’an, Shaanxi, China; 2 School of Information Science and Technology, Northwest University, Xi’an, Shaanxi, China; Victoria University, AUSTRALIA

## Abstract

The recommender system is widely used in the field of e-commerce and plays an important role in guiding customers to make smart decisions. Although many algorithms are available in the recommender system, collaborative filtering is still one of the most used and successful recommendation technologies. In collaborative filtering, similarity calculation is the main issue. In order to improve the accuracy and quality of recommendations, we proposed an improved similarity model, which takes three impact factors of similarity into account to minimize the deviation of similarity calculation. Compared with the traditional similarity measure, the advantages of our proposed model are that it makes full use of rating data and solves the problem of co-rated items. To validate the efficiency of the proposed algorithm, experiments were performed on four datasets. Results show that the proposed method can effectively improve the preferences of the recommender system and it is suitable for the sparsity data.

## 1 Introduction

With the rapid development of internet technology, the amount of information on the internet grows exponentially. To overcome the information overload problem [1], the recommender system (RS) [2, 3] has been widely used in our daily life to recommend information of the customer’s interest or provide personalized services based on the customer’s behavior data, which help the customer quickly obtain the required information from the mass data. Recently, RS has been successfully applied to a variety of fields, such as music [4], TV show [5], E-commerce [6], news [7], taxi [8], tourism [9], social media [10].

In RS, recommendations are made through different types of algorithms. As the core and key part of the RS, the recommendation algorithm [11, 12] determines the type and performance of the RS. In general, the recommendation algorithm is classified into four major categories:

- Demographic filtering approach [13], which is based on the assumption that customers with similar personal attributes (age, sex, etc.) may have similar preferences. The calculation is simple, and it is easy to achieve real-time response. Since the preferences of different customers with common personal attributes may be different, one of main problems of this method is the low reliability.
- Content-based filtering approach [14, 15], which utilizes user’s choices made in the past. Therefore, items that are similar to what the user has previously purchased or liked will be recommended.
- Collaborative filtering (CF) approach [16], where recommendations are made based on the user’s ratings of the items. Users with similar ratings are called nearest neighbors, if the nearest neighbors are found, the unrated items of the user are predicted through the neighbors, then, the RS recommends the items with high predicted ratings to the user.
- Hybrid filtering approach [17] which combines previous approaches using different knowledge sources to solve the problems existing in each one of these algorithms.

Among these recommendation approaches, CF is generally considered as one of the most used and most successful recommendation technologies in the RS, especially e-commerce websites such as Amazon.com, Netflix and Google News [18].

Collaborative filtering recommendation algorithms are usually classified into two classes: memory-based algorithms [19, 20] and model-based algorithms [21, 22]. The main difference is the processing of ratings. Memory-based algorithms include user-based collaborative filtering (UBCF) algorithms [20] and item-based collaborative filtering (IBCF) algorithms [23]. The UBCF algorithm focuses on obtaining the target user’s nearest neighbors and predicting his/her unrated items, conversely, the goal of the IBCF algorithm is items. In this paper, UBCF is taken to illustrate the improved method of similarity. The model-based algorithm [24] needs to build a model that represent users’ behavior according to the collected ratings, then, the unrated items can be predicted.

The value of similarity in CF mentioned above needs a similarity function to measure, some commonly used similarity metrics including cosine (COS), Pearson Correlation Coefficient (PCC), weighted Pearson Correlation Coefficient (WPCC) and Jaccard. Once the similarity method is chosen, recommendations can be made to users. Generally speaking, the above measures can well reflect the degree of similarity between two users or items. However, when the dataset is sparse, the accuracy of recommendation is very low. To solve this problem, plenty of similarity metrics have been proposed in recent years, nevertheless, the improvement is not obvious.

In this paper, our goal is to devise a similarity method that works for most recommender systems, regardless of the sparsity of the datasets. Based on the above considerations, this paper proposes three similarity impact factors to improve the accuracy and quality of the recommendations. Furthermore, the proposed similarity algorithm is normalized.

The structure of the paper is as follows. Section 2 reports some similarity calculation methods in the field of collaborative filtering recommendation. Section 3 describes the proposed similarity model in detail. Section 4 presents the experimental results in different datasets. Section 5 discusses the results and advantages of our proposed model. Finally, Section 6 gives a high level description of the conclusions and future work.

## 2 Related work

In this section, we briefly summarize the related work of similarity metrics. The larger the value, the higher the correlation. In the following formulas, we assume that the set of users and items are *U* = {*u*_1_, *u*_2_, ⋯, *u*_*m*_} and *I* = {*i*_1_, *i*_2_, ⋯, *i*_*n*_} respectively. *R* = [*r*_*ui*_]^*m*×*n*^ is used to represent the user-item rating matrix. Here, the number of users and items are *m* and *n* respectively, *r*_*ui*_ represents the rating made by user *u* on item *i*.

COS [25] similarity measures the angle between two rating vectors (users or items). Its similarity is frequently used in CF recommender system. The formula of COS similarity between user *u* and *v* is defined in Eq (1):

$$\text{Sim}(u,v{)}^{\text{COS}}=\frac{{\vec{r}}_{u}\cdot {\vec{r}}_{v}}{∥{\vec{r}}_{u}∥\cdot ∥{\vec{r}}_{v}∥}=\frac{\sum_{i\epsilon I_{u}\cap I_{v}}r_{ui}\cdot r_{vi}}{\sqrt{\sum_{i\epsilon I_{u}\cap I_{v}}r_{ui}^{2}}\cdot \sqrt{\sum_{i\epsilon I_{u}\cap I_{v}}r_{vi}^{2}}}$$ (1)

However, COS does not consider the user’s rating preference. In other words, some users tend to score high in general, while others prefer to give low, even if they like the items very much. Adjust cosine (ACOS) [26] similarity measure solves this problem by subtracting the average rating.

PCC [27] is defined on the set of co-rated items or users. WPCC [28] is based on PCC. The formulas of PCC and WPCC are described in Eqs (2) and (3) respectively. Intuitively, when the number of the co-rated items is less than the threshold in Eq (3), the similarity value is smaller than the result of PCC. On the contrary, if the number is the threshold or more, the similarity metric is still PCC. In experiments, the threshold is usually set to 50. Constrained Pearson Correlation (CPCC) [29] is a modified form of PCC, in which an absolute reference is used instead of the average rating. When the co-rated values are on the same side, the correlation can be increased. Another modified form of PCC is sigmoid function based on Pearson Correlation Coefficient (SPCC) [30] that weakens the similarity compared with PCC.

$$\text{Sim}(u,v{)}^{\text{PCC}}=\frac{\sum_{i\epsilon I_{u}\cap I_{v}}\left(r_{ui}-{\bar{r}}_{u})(r_{vi}-{\bar{r}}_{v}\right)}{\sqrt{\sum_{i\epsilon I_{u}\cap I_{v}}(r_{ui}-{\bar{r}}_{u}{)}^{2}}\cdot \sqrt{\sum_{i\epsilon I_{u}\cap I_{v}}(r_{vi}-{\bar{r}}_{v}{)}^{2}}}$$ (2)

$$\text{Sim}(u,v{)}^{\text{WPCC}}=\{\begin{array}{ll} \frac{\left|I_{u}\cap I_{v}\right|}{T}\cdot sim{\left(u,v\right)}^{PCC}, & if\;|I_{u}\cap I_{v}|<T\\ sim{\left(u,v\right)}^{PCC}, & otherwise \end{array}$$ (3)

In addition, Jaccard [31] is another popular measure used in CF. This measure only considers the number of items rated by two users instead of the ratings, which indicates the more co-rated items, the more similar. Therefore, the similarity metric is inaccurate in some cases. Different from Jaccard, Mean Squared Difference (MSD) [20] considers more about the absolute ratings. However, the application of this measure is not very wide. The formulas of Jaccard and MSD is shown in Eqs (4) and (5) respectively.

$$\text{Sim}(u,v{)}^{\text{Jaccard}}=\frac{\left|I_{u}\cap I_{v}\right|}{\left|I_{u}\cup I_{v}\right|}$$ (4)

$$\text{Sim}(u,v{)}^{\text{MSD}}=1-\frac{\sum_{i\epsilon |I_{u}\cap I_{v}|}(r_{ui}-r_{vi}{)}^{2}}{\left|I_{u}\cap I_{v}\right|}$$ (5)

To avoid the drawbacks of traditional measures, in [32], Bobadilla et al. came up with a method that combined Jaccard and Mean Squared Difference (JMSD), in which Jaccard is used to capture the proportion of the co-rated items and MSD is used to obtain the information of ratings. The formula of JMSD is expressed in Eq (6).

$$\text{Sim}(u,v{)}^{\text{JMSD}}=Sim(u,v{)}^{Jaccard}\cdot Sim(u,v{)}^{MSD}$$ (6)

In [33], Bobadilla et al. proposed another similarity method named MJD (Mean-Jaccard-Differences), which combined six similarity measures to obtain a global similarity. The weight of each measure was obtained through neural network learning. However, these two measures do not work in the case of sparse data.

Another classical method proposed by Ahn used three factors of similarity, namely Proximity, Impact and Popularity called PIP (Proximity-Impact-Popularity) [26]. Although PIP can alleviate the cold start problem, the disadvantages are still obvious. First, the similarity metric does not consider the absolute ratings, and it also ignores the proportion of the co-rated items. Second, the method does not consider each user’s global rating preference. Finally, the formula is not normalized and it is not convenient to combine with other methods. Based on the above considerations, Liu et al. proposed a new heuristic similarity model (NHSM) in [20]. This method is based on PIP and successfully overcomes the inadequacies of the PIP approach. The formula of NHSM is expressed in Eq (7).

$$\text{Sim}(u,v{)}^{\text{NHSM}}=Sim(u,v{)}^{JPSS}\cdot Sim(u,v{)}^{URP}$$ (7)

In [34], Polatidis et al. proposed a multi-level recommendation method to improve the quality of RS. This measure divides similarity into different levels and adds constrains to each level, the final similarity value depends on PCC and the number of co-rated items. The similarity metric adds a different constant to different level. The more co-rated items, the greater the constant.

Patra et al. proposed a new similarity measure using Bhattacharyya coefficient memory-based CF in sparse data, which used all ratings made by a pair of users in [35]. Beyond that, Zhang et al. proposed a novel data structure and designed linear algorithms to compute the similarities in [36], the final goal is to short the evaluation time and improve the efficiency of the development of RS. Moreover, Lee et al. introduced a preference model in [37], which is used to improve the accuracy of all existing CF algorithms. The preference model is obtained by maximum likehood estimation. On a recent work, Sun et al. proposed a new similarity measure of Triangle Multiplying Jaccard (TMJ) in [38], which combines triangle similarity and Jaccard similarly to improve recommendation accuracy. The TMJ is defined in Eq (8).

$$\text{Sim}(u,v{)}^{\text{TMJ}}=Sim(u,v{)}^{Triangle}\cdot Sim(u,v{)}^{Jaccard}$$ (8)

In summary, literature offers rich evidence on the successful performance of CF measures. However, the existing similarity method still has some limitations. First, CF measures suffer from serious data sparsity [39] and cold start [40] problems. In practice, the user-item rating matrix used for CF is extremely sparse and does not have enough ratings, so the performance of the CF recommender system is challenged by data sparsity. Cold start problem is an extreme situation that occurs when a new user or item just enters the system, and it is difficult to recommend for the lack of information. In the aforementioned issues, This paper focuses on the data sparsity problem. The main contribution of our work is that we propose a novel similarity model to minimize the deviation of similarity calculation and improve the accuracy of the recommendations, and our model can still maintain high recommendation accuracy in the case of data sparsity.

## 3 The proposed similarity model

This section first introduce the motivations of our proposed similarity model. Then, we give a description of the proposed similarity model in detail and analyze its time complexity. Finally, we present the prediction measure adapted in our work.

### 3.1 The motivations of the proposed similarity model

From the description of the previous section, we notice that the traditional CF methods heavily rely on the co-rated items. However, the similarity computation cannot be performed when there are no co-rated items, which is called co-rated items problem. Therefore, our novel model is proposed to solve this situation.

The motivations for our proposed similarity model lies in three aspects: First, our model takes all rated items into consideration, while, the traditional CF approaches only considers the co-rated items, which accounts for a small fraction of the rated items. Second, the proposed model can solve the co-rated items problem in datasets, even for the extremely sparse datasets. Third, the similarity model is not only decided by all the rated items, but also the user’s global preference.

### 3.2 Proposed algorithm

The recommendation algorithm in this paper provide users with recommendations through three steps: Initially, the ratings generated by the behavior of a user’s interactions are extracted and stored to the database. Then, the approach of k-nearest neighbors (KNN) [41] is applied to predict the ratings of the target user’s unrated items. The difficulty of KNN is how to calculate the similarities between the target user and his/her neighbors. An improved similarity model is proposed to minimize the deviation of similarity calculation and improve the accuracy of recommendation, which will be introduced below. Finally, the first *N* items with the top predicted ratings will be recommended to the target user.

The main part of memory-based CF method is similarity calculation, which can be calculated either on pair of users or items. To evaluate the proposed similarity algorithm, UBCF is adapted in this paper. In order to improve the adaptability of the similarity metric in the case of the sparse rating data, the proposed similarity model is composed of three impact factors including *S*_1_, *S*_2_ and *S*_3_. Additionally, *S*_1_ is used to define the similarity between users. *S*_2_ is introduced to punish the user pairs with small proportion of the number of co-rated items. *S*_3_ is adopt to weight each user’s rating preference. The framework is defined in Eq (9).

$$\text{Sim}(u,v{)}^{\text{Proposed}}=S_{1}(u,v)\cdot S_{2}(u,v)\cdot S_{3}(u,v)$$ (9)

The similarity *S*_1_ is defined to measure the angle between the rating vectors of two users. The smaller the angle, the higher the degree of the similarity between the two users. Different from the traditional COS similarity method, *S*_1_ converts the angle calculation problem from the original |*I*_*u*_ ∩ *I*_*v*_| dimension space to |*I*_*u*_ ∪ *I*_*v*_| dimension space. That is, the calculation converts from the set of two users’ co-rated items to the union of two users’ rated items, which makes the rating data fully utilized. However, the sparsity of the dataset in the RS directly determines the accuracy of the angle calculation. If the sparsity of the dataset is low, the similarity between two users is calculated based on all existing rating data. In contrast, if the sparsity is high, there is almost no co-rated items between any two users, and the traditional similarity method does not work. In this case, we construct co-rated items in the new rating space, and replace the ratings of the unrated items with the average ratings to improve the accuracy of the algorithm. Based on the sparsity of dataset, *S*_1_ is divided into to two levels. The formula of *S*_1_ is defined in Eq (10). From the formula, it can be seen that the denominator has become larger compared with the traditional COS measure.

$$\begin{array}{c} \begin{array}{cc} S_{1}(u,v) & =\{\begin{array}{cc} \frac{\sum_{i\epsilon I_{u}\cap I_{v}}r_{ui}\cdot r_{vi}}{\sqrt{\sum_{i\epsilon I_{u}}r_{ui}^{2}}\cdot \sqrt{\sum_{i\epsilon I_{v}}r_{vi}^{2}}} & if\;sparsity<\rho \\ \frac{\sum_{i\epsilon I_{u}\cup I_{v}}r_{ui}\cdot r_{vi}}{\sqrt{\sum_{i\epsilon I_{u}\cup I_{v}}r_{ui}^{2}}\cdot \sqrt{\sum_{i\epsilon I_{u}\cup I_{v}}r_{vi}^{2}}} & otherwise \end{array}\\ & =\{\begin{array}{cc} \frac{\sum_{i\epsilon I_{u}\cap I_{v}}r_{ui}\cdot r_{vi}}{\sqrt{\sum_{i\epsilon I_{u}}r_{ui}^{2}}\cdot \sqrt{\sum_{i\epsilon I_{v}}r_{vi}^{2}}} & if\;sparsity<\rho \\ \frac{\sum_{i\epsilon I_{u}\cap I_{v}}r_{ui}\cdot r_{vi}+\sum_{i\epsilon I_{u}-I_{u}\cap I_{v}}r_{ui}\cdot \mu_{v}+\sum_{i\epsilon I_{v}-I_{u}\cap I_{v}}\mu_{u}\cdot r_{vi}}{\sqrt{\sum_{i\epsilon I_{u}}r_{ui}^{2}+\sum_{i\epsilon I_{u}\cup I_{v}-I_{u}}\mu_{u}^{2}}\cdot \sqrt{\sum_{i\epsilon I_{v}}r_{vi}^{2}+\sum_{i\epsilon I_{u}\cup I_{v}-I_{v}}\mu_{v}^{2}}} & otherwise \end{array} \end{array} \end{array}$$ (10)

Where *μ*_*u*_ and *μ*_*v*_ are the average ratings of user *u* and user *v* respectively. In the case of sparsity less than the threshold *ρ*, the similarity is calculated in |*I*_*u*_ ∪ *I*_*v*_| dimension space. In the new rating space, zero is used to indicate the user’s rating of the unrated items. Unlike the traditional COS similarity method, which only uses the rating data on the co-rated items, our proposed similarity *S*_1_ uses the two users’ entire rating data. In other cases, the ratings of the unrated items in this space are replaced with the users’ average ratings, and then calculate the similarity.

In the recommender system, the number of co-rated items between different user pairs varies greatly. The more co-rated items, the more valuable information is extracted from the rating data, and the similarity calculation result will be more accurate. Consequently, the proportion of the number of co-rated items is a very important impact factor, and its definition is in Eq (11). If the proportion of the co-rated items is small, the value of *S*_2_ will be low.

$$S_{2}(u,v)=\frac{1}{1+\exp(-\frac{(I_{u}\cap I_{v}{)}^{2}}{\left|I_{u}|\cdot |I_{v}\right|})}$$ (11)

In our model, *S*_3_ is used to indicate the rating preference of each user. Due to different users have different rating habits, some users like to give high ratings, while others may prefer rating low. Therefore, the rating preference should be considered. *S*_3_ is adopted to revise our proposed model. The formula of *S*_3_ is defined in Eq (12) [19], which is determined by average rating and standard variance.

$$S_{3}(u,v)=1-\frac{1}{1+\exp(-|\mu_{u}-\mu_{v}|\cdot |\delta_{u}-\delta_{v}|)}$$ (12)

Where |⋅| returns the absolute value of the function. *δ*_*u*_ and *δ*_*v*_ represent the standard variance of user *u* and user *v*.

Thus, our proposed similarity model is divided into two levels based on the data sparsity of the RS, the similarity between user *u* and *v* is the product of *S*_1_, *S*_2_ and *S*_3_. Compared with the traditional CF methods, the advantages of our proposed model are: First, more data not just the co-rated items is used in the similarity factor of *S*_1_, to extract more useful information. Second, from the defined formulation of *S*_1_, we notice that the proposed model completely solves the problem of co-rated items. Consequently, it broadens the scope of application of the traditional memory-based CF approaches, even for the sparse data. Third, we not only consider the influence the co-rated items (factor *S*_2_), but also take into account the impact of the global preference of user’s behavior (factor *S*_3_).

### 3.3 Time complexity

According to the assumptions in section 2, the number of users and items are *m* and *n* respectively. From the definition of our proposed similarity model we can see that its time complexity of user similarity computation is *O*(*n*). In this paper, KNN is adopted to find each user’s nearest neighbors. Hence, the time complexity of finding all neighbors is *O*(*mn*).

Since the maximum number of ratings in the dataset is *mn*, all unrated items should be predicted by the proposed model, therefore, the overall time complexity for the whole dataset is *O*(*m*^2^*n*^2^).

### 3.4 Ratings prediction

If the similarities are prepared, the ratings of unrated items can be predicted. In this paper, the prediction formula [26] of a rating of user *u* on item *i* is expressed in Eq (13).

$$p_{\text{ui}}={\bar{r}}_{u}+\frac{\sum_{v\epsilon NN_{u}}Sim(u,v)\cdot (r_{vi}-{\bar{r}}_{v})}{\sum_{v\epsilon NN_{u}}|Sim(u,v)|}$$ (13)

Where *NN*_*u*_ indicates the set of nearest neighbors of user *u*. Based on the above method, all unrated items of the target user can be calculated. Then, the RS recommends the top *N* items to the target user as the recommendation results.

## 4 Experiments

In this section, we first carry out a set of sparsity experiments to determine the optimal threshold *ρ* in our proposed model. Then, to verify the superiority of the proposed similarity model based on CF, we conduct experimental evaluation on four real datasets and compare our model with other methods of CF including COS, PCC, WPCC, Jaccard, MSD, JMSD, NHSM and TMJ, which are described in the related work section.

### 4.1 Datasets

Four datasets of Movielens 100K, FilmTrust, Ciao and Epinions are employed to evaluate the effectiveness of our algorithm. Because these four datasets are often used by researchers to verify the performance of CF recommendation algorithms, and their sparsity is quite different, so we choose the above four datasets. In this paper, we assume that sparsity is the ratio of the number of unrated user-item pairs to the total number of user-item pairs in the user-item rating matrix. Moreover, the sparsity in Eq (10) refers to the sparsity of the training datasets.

- Movielens 100K dataset (http://grouplens.org/datasets/movielens) contains 100,000 ratings of 1,682 movies made by 943 users. In this dataset, each user has rated at least 20 movies. All the rating values are integer in the scale 1 to 5, where rate 1 shows that the user is not interested in the movie, and rate 5 means that the user favors the movie very much. The sparsity of Movielens 100K is 93.7%.
- FilmTrust dataset is a small and publicly available dataset extracted from the entire FilmTrust website. The dataset has 35,497 ratings from 1,508 users on 2,071 movies, and the rating value is a multiple of 0.5, ranging from 0.5 to 4. The sparsity of FilmTrust is 98.86%.
- Ciao dataset is a publicly available dataset retrieved from the entire DVD category on the dvd.co.uk website. It contains 72,665 ratings from 17,615 users on 16,121 movies and all the rating values are integer in the scale 1 to 5. The sparsity of Ciao is 99.9744%.
- Epinions dataset (http://www.trustlet.org/epinions.html) is a publicly available and general product recommendation dataset. The dataset contains 664,823 ratings of 139,738 items rated by 40,163 users. The rating range in the dataset is an integer value from 1 to 5. Moreover, the sparsity of Epinions is 99.9882%.

To demonstrate the performance of the proposed similarity measure, each dataset is divided into two parts, 80% randomly selected of part is used for training, and the remaining 20% is used for testing. Hence, the sparsity of the above four training datasets are 94.956%, 99.09%, 99.98%, and 99.99% respectively.

### 4.2 Evaluation metrics

To estimate the performance of RSs, the mean absolute error (MAE), rooted mean squared error (RMSE) [42], precision and recall are among the most popular ones. According to [43], the metrics evaluating recommendation systems can be roughly classified into two categories: prediction accuracy and classification accuracy. MAE and RMSE are mainly used to evaluate the prediction accuracy [36], while precision and recall are used to evaluate the quality of top-N recommendation [20]. In this paper, we adopt the metrics of MAE and RMSE to represent the accuracy of the proposed algorithm.

MAE is the most used metric in collaborative filtering RS, which is used to estimate the average absolute deviation between the actual ratings and the prediction ratings, MAE is defined in Eq (14).

$$\text{MAE}=\frac{1}{TN}\sum_{u\epsilon U,i\epsilon I}|p_{ui}-r_{ui}|$$ (14)

Where *TN* is the total number of items in the set. *p*_*ui*_ and *r*_*ui*_ represent the predicted rating and actual rating of user *u* on item *i* respectively. The smaller the metric, the higher the prediction accuracy. However, MAE is not normalized.

RMSE reflects the degree of deviation between the predicted ratings and the actual ones, which penalizes large deviation more heavily for squaring the errors before summing them. Lower RSME corresponds to higher prediction accuracy. RMSE is evaluated in Eq (15).

$$\text{RMSE}=\sqrt{\frac{1}{TN}\sum_{u\epsilon U,i\epsilon I}(p_{ui}-r_{ui}{)}^{2}}$$ (15)

### 4.3 The threshold *ρ*

We remind that the threshold *ρ* is a policy factor affecting the results of the proposed model. Before the experimental evaluation process, we set the threshold in the proposed model to the extremes of *ρ* = 1 and *ρ* = 0 respectively, in which case the model is converted from two levels (level 1 and level 2) to one level. When *ρ* = 1, the remaining level is called level 1. Similarly, *ρ* = 0 corresponds to level 2. In this section, our research focuses on the effects of sparsity on the two different levels to further determine the optimal threshold. Moreover, the performance of the recommendation is estimated by the metrics of MAE and RMSE. In CF algorithms, since the number of nearest neighbors also affects the accuracy and quality of recommendation, it is set to a fixed value of 30 in this set of experiments on sparsity.

**Fig 1:**
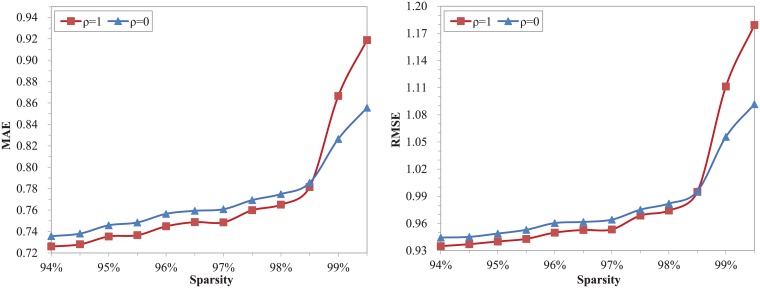
The performance of the proposed model at two extreme thresholds with different sparsity.

In Fig 1, we illustrate our results of the proposed model on the conditions of *ρ* = 1 and *ρ* = 0 with the values of MAE and RMSE changed by sparsity. Datasets with different sparsity levels are constructed by changing the proportion of the training set in the Movielens 100K. Sparsity varies from 94% to 99.5%, and the step size is 0.5%. From the figure we see that the performance of our model continually deteriorates under two threshold conditions with the increasing of sparsity, especially when the sparsity is greater than 98.5%. It demonstrates that sparsity is an important factor affecting the accuracy of recommendation, and the higher the sparsity, the greater the impact. When the sparsity is less than 98.5%, our proposed model on the condition of *ρ* = 1 has lower MAE and RMSE, contrarily, the model on the condition of *ρ* = 0 has better metrics when the sparsity is greater than 99%. As *ρ* = 1 corresponds to the level 1, and *ρ* = 0 corresponds to the level 2, the figure clearly show that the level 1 surpasses the level 2 when the sparsity is less than 98.5%, however, the level 2 has better performance when the sparsity is greater than 99%. Consequently, we set the threshold *ρ* = 0.985 in the following experiments to ensure that the proposed model performs better in the whole sparsity range.

### 4.4 Settings

In order to evaluate the effectiveness of the proposed similarity model, we compare our similarity algorithm with some other measures by using the metrics of MAE and RMSE in four datasets. As is known, the performance of the recommendation algorithms is affected by the number of nearest neighbors, which is denoted by K in this paper. Considering the recommendation efficiency and accuracy of the recommendation algorithm, K varies from 5 to 100, and the step size is 5. Besides, the threshold *ρ* in Eq (10) is set to 0.985. For the below experiment evaluation, the experiment results of our proposed method are compared with the other eight CF recommendation algorithms under the conditions of different K.

### 4.5 Experimental results and analysis

**Fig 2:**
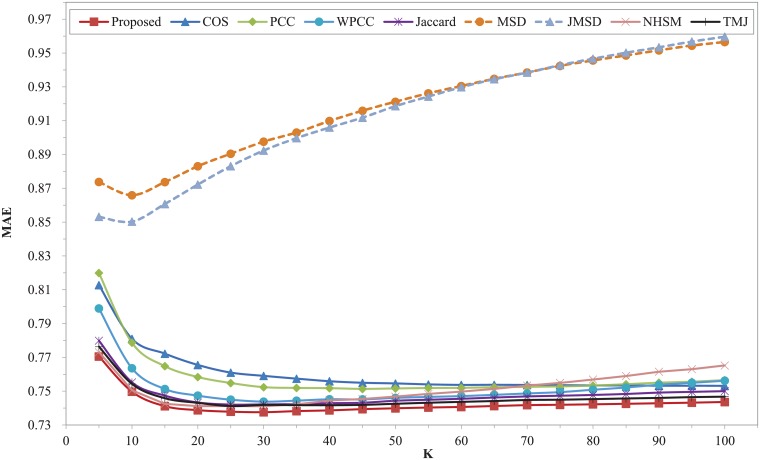
MAE of different similarity measures with different K on Movielens 100K dataset.

**Fig 3:**
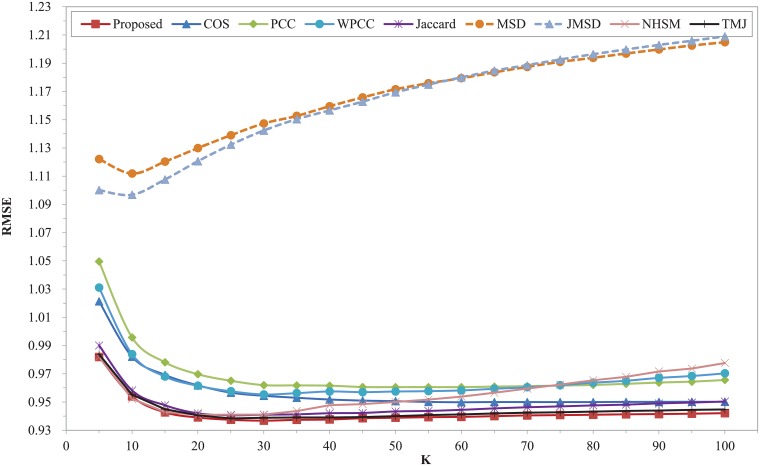
RMSE of different similarity measures with different K on Movielens 100K dataset.

Figs 2 and 3 show the MAE and RMSE of different similarity measures with different K on the dataset of Movielens 100K respectively. Both MAE and RMSE firstly decrease with the increasing of nearest neighbors and then increase slightly for different similarity measures except COS. The two curves of COS measure decrease within the range of K. The two plots clearly shows that our proposed model surpasses other measures over the entire range of K, while, the two measures of MSD and JMSD are apparently inferior to other similarity measures. Our measure has the best MAE and RMSE when K = 30. The performance of TMJ is closest to our proposed model. It can be observed that the classic measures of COS, PCC, WPCC, MSD and JMSD exhibit larger values of MAE and RMSE in the whole range. The MAE and RMSE of NHSM measure simultaneously reach the lowest point when K is 20, which increases significantly with the increase of K when it is greater than 20. The Jaccard measure owns a good result in this dataset. Moreover, our proposed model is more stable than other measures throughout the nearest neighbors.

**Fig 4:**
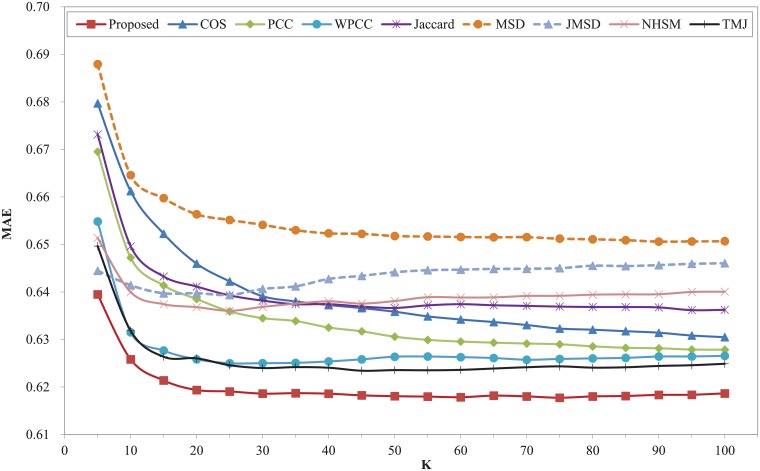
MAE of different similarity measures with different K on FilmTrust dataset.

**Fig 5:**
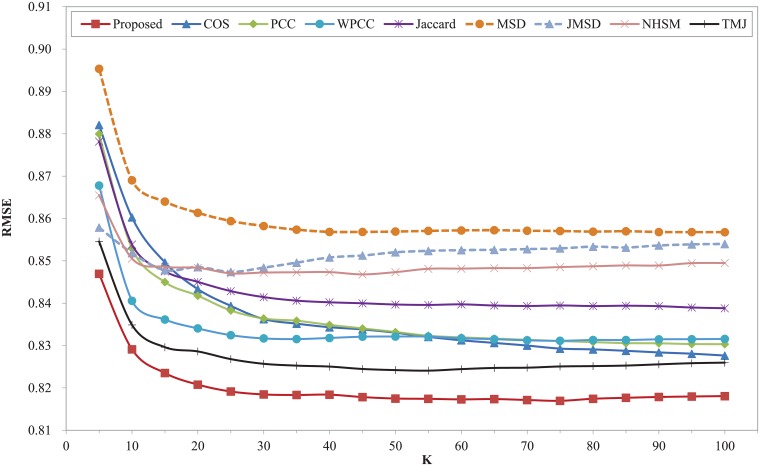
RMSE of different similarity measures with different K on FilmTrust dataset.

The nine different similarity measures are executed on FilmTrust dataset. The prediction errors of MAE and RMSE with different number of nearest neighbors are shown in Figs 4 and 5. Compared with the recently proposed measure of TMJ, the improvement of our proposed model is remarkable. The two figures clearly show that our proposed measure outperforms all other measures, and it has the best results of MAE and RMSE when K = 75. In this dataset, the MSD measure has the worst MAE and RMSE over the entire range of K. The performance of TMJ measure ranks second in the whole range. The classic measures of COS, PCC, WPCC, Jaccard, JMSD and NHSM have worse results in these two metrics. Moreover, the WPCC measure is better than PCC when K is less than 70. Compared with TMJ, our proposed model has at least 1.56% and 0.96% higher improvement in terms of the MAE and RMSE respectively. Compared with COS, our proposed model has at least 7.04% and 5.4% higher improvement in terms of the MAE and RMSE respectively.

**Fig 6:**
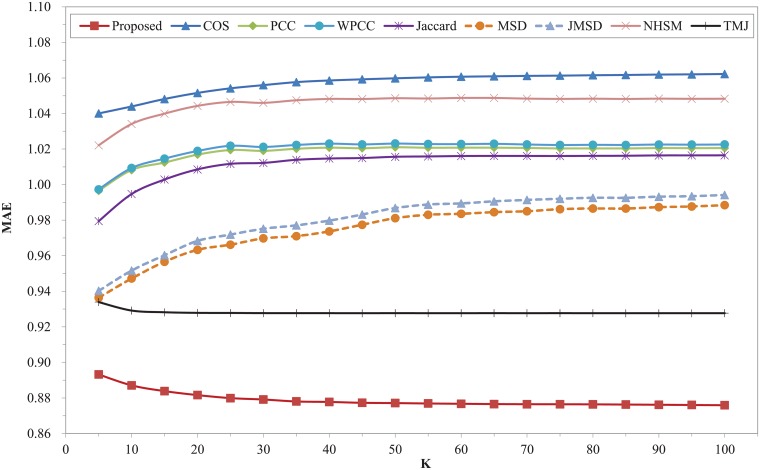
MAE of different similarity measures with different K on Ciao dataset.

**Fig 7:**
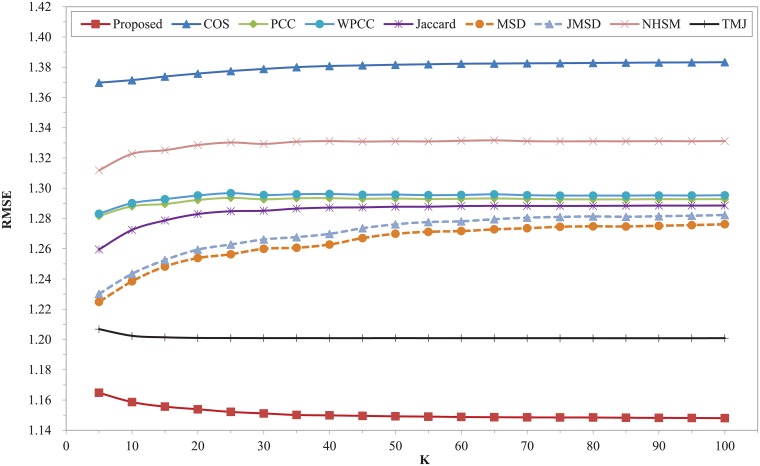
RMSE of different similarity measures with different K on Ciao dataset.

Figs 6 and 7 show the prediction errors of nine similarity measures on the Ciao dataset. It can be seen that our proposed model is apparently superior to other measures over the entire range of K in terms of MAE and RMSE, and the curves of our proposed model descend slowly over the entire K value range. The MAE and RMSE of some classic similarity measures including COS, PCC, WPCC, Jaccard, MSD, JMSD, NHSM are comparatively high. Especially the COS similarity measure, which obtains the worst results in the whole range. The recently proposed similarity measure of TMJ obtains a good result of MAE and RMSE. Compared with TMJ, the MAE and RMSE of our proposed model reduce at least 5.58% and 4.4% respectively. Compared with COS measure, the MAE and RMSE of our proposed model reduce at least 17.54% and 17% respectively. Furthermore, the performance of all measures is relatively stable throughout the nearest neighbors.

**Fig 8:**
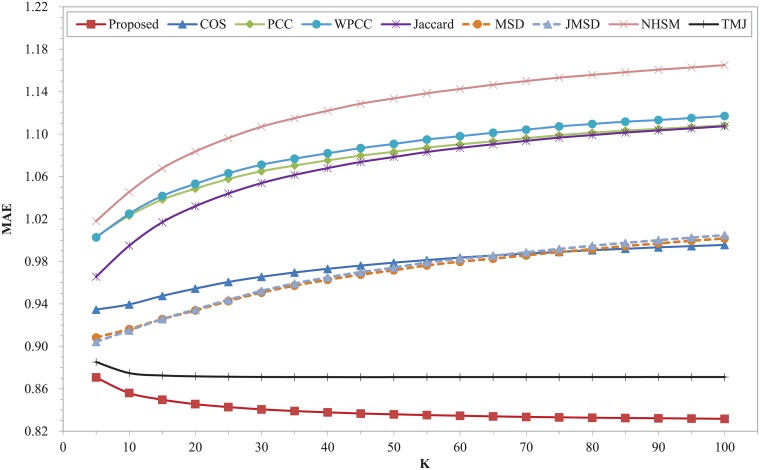
MAE of different similarity measures with different K on Epinions dataset.

**Fig 9:**
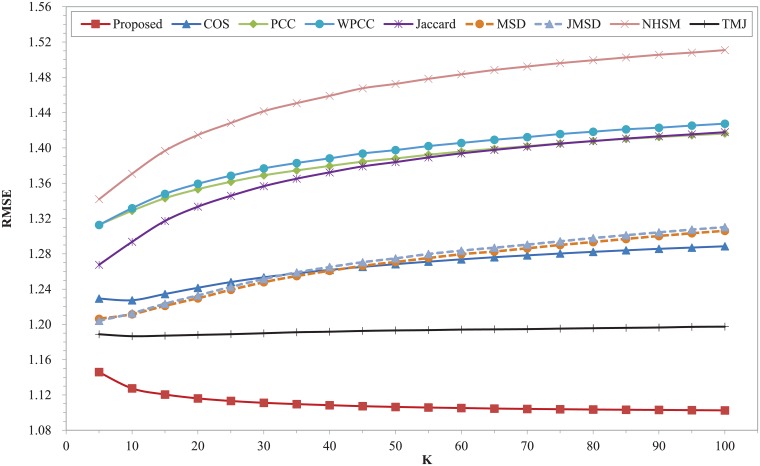
RMSE of different similarity measures with different K on Epinions dataset.

Finally, the prediction errors of the nine CF measures are tested on Epinions dataset. The results of MAE and RMSE with different K are shown in Figs 8 and 9. It can be seen that our proposed model obtains best results in the whole range compared with all other measures. The MAE and RMSE of our measure decrease with the increasing of K, while the measures results of COS, PCC, WPCC, Jaccard, MSD, JMSD and NHSM increase with the increasing of K. In this dataset, the NHSM measure owns the worst results in the whole range. The classic measures of COS, PCC, WPCC, Jaccard, MSD and JMSD have worse results in these two metrics. Compared with PCC, the WPCC measure achieves worse results over the entire range of K. The performance of the TMJ measure is stable and ranks second. Compared with TMJ, the MAE and RMSE of our proposed model reduce at least 4.5% and 7.9% respectively. Compared with COS measure, the MAE and RMSE of our proposed model reduce at least 28.6% and 27% respectively.

## 5 Discussions

In this paper, a novel similarity model is proposed, which is constructed of three impact factors, *S*_1_, *S*_2_ and *S*_3_. *S*_1_ defines the similarity between users. It is divided into two levels based on sparsity of the dataset in the RS to improve accuracy and ensure efficiency. *S*_2_ is used to punish the small proportion of co-rated items. User’s rating preference is weighted by *S*_3_.

The experiments are implemented on four datasets with different sparsity levels, and the sparsity of the four training datasets (Movielens 100K, FilmTrust, Ciao, Epinions) is 94.956%, 99.09%, 99.98%, and 99.99% respectively. The sparsity levels are constantly increasing. Our proposed model is compared with other eight measures such as COS, PCC, WPCC, Jaccard, MSD, JMSD, NHSM and TMJ in two metrics of MAE and RMSE. The experiment results show that our measure achieves better performance on four datasets compared with all other measures, especially on extremely sparse datasets. Compared with the recently proposed measure of TMJ, although the sparsity of the Movielens 100K is the lowest, the advantage of our model is not obvious. On other three datasets, our model achieves remarkable improvement. Compared with all other measures, the MAE and RMSE of our proposed model reduce 1.56%—7.04% and 0.96%—5.4% respectively on FilmTrust dataset, the MAE and RMSE of our proposed model reduce at least 5.58% -17.54% and 4.4%—17% respectively on Ciao dataset, and the MAE and RMSE of our proposed model reduce at least 4.5%—28.6% and 7.9%—27% respectively on Epinions dataset. From the data analysis, it is seen that the advantages of our model are more pronounced with the increasing of sparsity. In addition, the performance of our model is relatively stable on all four datasets.

Therefore, the experiment results verify that effectiveness of our proposed similarity model, and it is more suitable for RS especially on extremely sparse recommender systems.

## 6 Conclusions and future work

In this paper, we concentrate on improving the preference and quality of recommendation in case of sparsity data. To alleviate this problem, an improved similarity model is proposed. Three similarity impact factors were taken into account in the proposed model. To validate the effectiveness of the proposed measure, we employed four popular datasets of Movielens 100K, FilmTrust, Ciao, Epinions. Results suggest that the proposed similarity measure can effectively improve the accuracy of recommendation when the data is sparsity, and it can overcome the drawbacks of the traditional similarity measures.

The academic contribution of our work can be summarized as follows. First, our model make full use of the rating data to improve the accuracy of recommender systems. All the rating data from users is used in the model, not just the co-rated rating data. Second, the problem of co-rated items is solved in our model, which still can obtain an accurate similarity when there is no co-rated items between two users. Third, this paper proposes a new similarity model for collaborative filtering approaches, which shows superior performance than the traditional similarity measures such as COS, PCC, WPCC, Jaccard, MSD, JMSD and NHSM. Finally, most studies that alleviate the data sparse problem have designed more complex models or utilized additional content-based information, which will increase the calculation time. Our purpose is to improve the existing traditional similarity measure just based on available rating data, and the proposed measure can be regarded as a substitute for the traditional measures.

However, the proposed similarity measure still suffers from the complete cold start problem. In our future research issues, inspired by one class of collaborative filtering approaches, we plan to adapt a Matrix Factorization framework to address the new user complete cold start problem and further improve the accuracy of the recommendation.
