# Correction: An improved collaborative filtering method based on similarity

**DOI:** 10.1371/journal.pone.0206629

**Published:** 2018-10-24

**Authors:** The PLOS ONE Staff

The second author's name is spelled incorrectly. The publisher apologizes for the error. The correct name is: Xiaoyi Feng. The correct citation is: Feng J, Feng X, Zhang N, Peng J (2018) An improved collaborative filtering method based on similarity. PLoS ONE 13(9): e0204003. https://doi.org/10.1371/journal.pone.0204003
